# Adaptive Feature- and Scale-Based Object Tracking with Correlation Filters for Resource-Constrained End Devices in the IoT

**DOI:** 10.3390/s25165025

**Published:** 2025-08-13

**Authors:** Shengjie Li, Kaiwen Kang, Shuai Zhao, Bo Cheng, Junliang Chen

**Affiliations:** State Key Laboratory of Networking and Switching Technology, Beijing University of Posts and Telecommunications, Beijing 100876, China; kkw0482@outlook.com (K.K.); zhaoshuaiby@bupt.edu.cn (S.Z.); chengbo@bupt.edu.cn (B.C.); chjl@bupt.edu.cn (J.C.)

**Keywords:** visual object tracking, discriminative correlation filter, adaptive mapped feature and scale interval, resource-constrained end devices, Internet of Things

## Abstract

Sixth-generation (6G) wireless technology has facilitated the rapid development of the Internet of Things (IoT), enabling various end devices to be deployed in applications such as wireless multimedia sensor networks. However, most end devices encounter difficulties when dealing a large amount of IoT video data due to their lack of computational resources for visual object tracking. Discriminative correlation filter (DCF)-based tracking approaches possess favorable properties for resource-constrained end devices, such as low computational costs and robustness to motion blur and illumination variations. Most current DCF trackers employ multiple features and the spatial–temporal scale space to estimate the target state, both of which may be suboptimal due to their fixed feature dimensions and dense scale intervals. In this paper, we present an adaptive mapped-feature and scale-interval method based on DCF to alleviate the problem of suboptimality. Specifically, we propose an adaptive mapped-feature response based on dimensionality reduction and histogram score maps to integrate multiple features and boost tracking effectiveness. Moreover, an adaptive temporal scale estimation method with sparse intervals is proposed to further improve tracking efficiency. Extensive experiments on the DTB70, UAV112, UAV123@10fps and UAVDT datasets demonstrate the superiority of our method, with a running speed of 41.3 FPS on a cheap CPU, compared to state-of-the-art trackers.

## 1. Introduction

With the recent breakthroughs in wireless technology, the sixth-generation (6G) wireless network could play a key role in the development of the Internet of Things (IoT), where a wide variety of end devices have been deployed to monitor data flows, collect resources and supervise this large-scale, complex network [1]. With the growing interest in positioning and tracking using end devices in wireless multimedia sensor networks, visual object tracking has drawn considerable attention due to its wide range of IoT applications, such as smart transportation, wildlife monitoring and military surveillance [2]. A challenging in visual tracking is developing a robust algorithm that can detect and locate objects in scenarios with partial occlusion, motion blur, illumination variation, background clutter and scale variations [3,4].

Briefly, there are two major categories of visual tracking approaches: discriminative correlation filter (DCF)-based trackers [5] and deep learning-based trackers [6,7,8]. Benefiting from a large amount of offline training data and high-end GPU resources, deep learning-based methods have achieved impressive tracking performance and can determine the semantic features of the target at a high level [9,10]. However, these trackers implemented on expensive GPUs are impractical for most end devices, as they can generally only be equipped with a common CPU. Moreover, the stringent real-time requirements for end devices (e.g., UAVs) in some IoT applications exacerbate these difficulties [11]. Therefore, it is crucial to explore CPU-based robust visual tracking methods suitable for resource-constrained end devices in various IoT applications.

Thanks to the assumption that training samples are periodic and the use of the fast Fourier transform (FFT) in the frequency domain, DCF trackers have the advantage of being computationally efficient, making them especially suitable for resource-constrained end devices in real-time IoT applications. MOSSE [12] is one notable example, running about 700 frames per second (FPS) with an adaptive correlation filter. Along with the introduction of kernelized correlation filters [13], scale estimation [14,15], multiple-feature representations [16,17] and boundary effect suppression [18,19], DCF trackers have achieved performance improvements in terms of both accuracy and robustness. However, these improvements have resulted in decreased computational efficiency. Most state-of-the-art DCF methods with multiple features and a dedicated scale-space filter in every frame require a gradual increase in running time to achieve accurate tracking results and are not as suitable for real-time IoT applications as early DCF trackers, where the speed of the DCF [13] is ∼292 FPS on a CPU.

**Figure 1:**
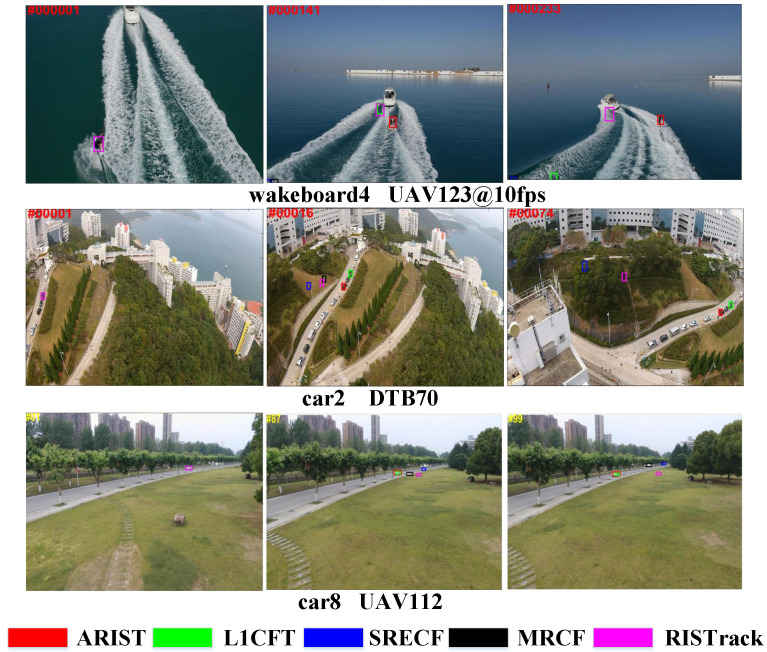
Tracking results of ARIST compared to four state-of-the-art (SOTA) methods for real-time tracking in UAV123@10fps [4], DTB70 [21] and UAV112 [3]. ARIST, adopting an adaptive mapped feature and temporal scale based on RISTrack [20], achieves more accurate results and performs favorably when compared to four SOTA DCF-based trackers (L1CFT [11], SRECF [19], MRCF [14] and RISTrack [20]).

To enable a trade-off between effectiveness and efficiency, a response-interference-suppression correlation filter (RISTrack) [20] is proposed to achieve competitive performance compared with state-of-the-art methods. Given an image patch, RISTrack extracts multi-channel features from the target region and efficiently solves the problem of constrained correlation filter learning in the Fourier domain using ADMM. To improve temporal consistency and robustness, a response-interference-suppression mechanism is employed to ensure consistency across consecutive responses and suppress distractor responses. Additionally, a response auxiliary strategy is applied to smooth the response, enhancing localization stability. The improvement in RISTrack is attributed to its ability to suppress background distractors and maintain temporal consistency of the responses using historical information. Although superior in terms of accuracy and robustness in real time, RISTrack still needs to improve its overall tracking capability. For example, in various in IoT application challenges, RISTrack exhibits inferior performance, as shown in Figure 1, because it applies fixed feature dimensions and dense scale intervals to estimate the target state.

In this paper, we propose an adaptive mapped-feature and scale-interval approach employed in RISTrack for real-time object tracking. Specifically, an adaptive mapped-feature response is proposed based on the dimensionality reduction strategy, which assigns histogram score maps to adaptive-dimension features to generate the final responses, and multiple features are employed to effectively achieve robust target-state estimation. Meanwhile, we present an efficient scale estimation method using the adaptive temporal scale interval instead of densely sampling the spatial–temporal scale space to overcome various challenges. Our experimental evaluations demonstrate that the proposed method exhibits significant performance improvement when compared to the baseline and state-of-the-art trackers. Figure 1 shows a comparison of our method (ARIST) against state-of-the-art methods on three videos from the UAV123@10fps [4], DTB70 [21] and UAV112 [3] datasets. To summarize, the main contributions of this work are three-fold:

- We propose an adaptive mapped-feature and scale-interval method with a response-interference-suppression correlation filter for real-time object tracking, which adopts fewer feature dimensions and sparse temporal scale intervals while maintaining high accuracy.
- To improve upon existing feature integration and scale estimation methods, two adaptive methods are proposed for temporal scale intervals and mapped-feature responses based on dimensionality reduction and histogram scores to boost overall performance.
- Extensive experiments are performed on the UAV123@10fps [4], DTB70 [21], UAV112 [3] and UAVDT [22] datasets. The proposed tracker performs favorably compared to state-of-the-art trackers, with a real-time tracking speed of 41.3 FPS on a cheap CPU.

The proposed adaptive mapped-feature and scale-interval method can be used for many DCF trackers based on its handcrafted features and dense temporal scale intervals. Furthermore, our tracker can achieve better results by adopting more sophisticated baselines and has considerable potential for future improvement. However, the implemented ARIST is far from optimal. The rest of this paper is organized as follows: A review of relevant studies is presented in Section 2. In Section 3, we review RISTrack and introduce our adaptive mapped-feature response and adaptive temporal scale-interval estimation method. In Section 4, we present our experimental results and compare our method with the RISTrack method and state-of-the-art methods. Finally, we present our conclusions in Section 5.

## 2. Related Work

Visual object tracking has long been a popular research problem, with extensive studies [2] conducted over the past few years. In this section, we mainly review the existing DCF-based visual tracking approaches.

Recently, DCF-based trackers have been successfully applied for visual tracking [5,19,23]. As the motion and discriminative models of these methods are coupled, high-speed tracking can be achieved by regressing all of the circular-shifted samples about the input features into soft labels from the Gaussian distribution and converting the correlation in the spatial domain to an element-wise product in the Fourier domain. Bolme et al. [12] trained an adaptive correlation filter by learning the minimum output sum of the squared error in the luminance channel. Henriques et al. [13] proposed fast kernelized correlation filters by minimizing the loss function of ridge regression and further extended this fast algorithm to the multiple-channel HOG features. Danelljan et al. [23] then explored color space features based on kernelized correlation filters. These approaches have achieved excellent performance on tracking-benchmark datasets [3,22], which run in real time. Several studies have been conducted to assess the effectiveness of these popular trackers for robust visual tracking. For example, power features such as deep convolution features have been used [8,9], and to cope with the boundary effect, spatial regularization [24] and limited boundaries [14,17,25] have also been investigated. In addition, sparse regularization [11], continuous convolution [26], efficient operators [18] and dynamic distractor-repression [16] have also been developed to improve tracking performance.

Although superior tracking performance has been achieved by these methods, most deep learning methods suffer from high computational complexity, even with expensive GPUs, and cannot be used in real-time IoT applications. To overcome this problem, a response-interference-suppression correlation filter with multiple-channel HOGs and CN features [20] is proposed to suppress background distractors and maintain the temporal consistency of the responses, improving performance and enabling real-time IoT application. However, this approach requires extra calculation in the target-state estimation stage, where multiple features and the spatial–temporal scale space are employed with fixed feature dimensions and dense scale intervals. Contrary to [20], we aim to estimate the target state with the response-interference-suppression correlation filter using adaptive mapped-feature responses and temporal scale estimation, which will allow us to achieve fast and robust tracking with fewer feature dimensions and sparse scale intervals.

## 3. Proposed Algorithm

**Figure 2:**
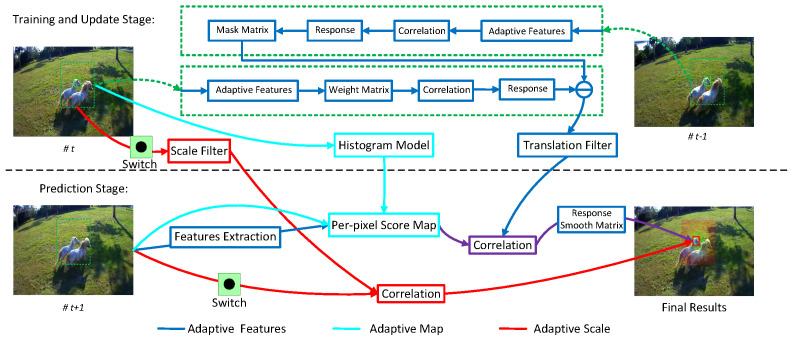
Flowchart of our approach with adaptive mapped features and scale intervals for real-time tracking.

A flowchart of the proposed algorithm is shown in Figure 2. The green dotted line represents the target templates; the green solid line represents the previous target box; the blue solid line represents the adaptive feature based on dimensionality reduction; the cyan solid line represents the adaptive map based on the histogram-based per-pixel score; the purple solid line indicates the adaptive mapped-feature responses; and the red solid lines represent the predicted target boxes caused by the adaptive temporal scale-interval estimation. These pipelines are integrated into the processes of adaptive feature mapping and scale estimation for more accurate object predictions. Furthermore, we base our approach on RISTrack, which achieves very promising results on popular datasets [20]. Although RISTrack simply adapts handcrafted features, it achieves competitive performance compared to deep learning-based trackers [3,4,21,22]. The core idea of RISTrack is that the background distractor suppression and historical guidance are augmented to improve the discriminative ability of the learned filter while maintaining a closed-form solution for tracking efficiency. In the following section, we briefly review the main features of RISTrack.

### 3.1. Response-Interference-Suppression Correlation Filter Tracking

Using RISTrack, Yan Li et al. [20] proposed a correlation filter-based framework, based on the conventional STRCF [24], that provided a basic foundation for learning correlation filters with spatial regularization. Building upon this, the proposed method introduces a response-interference-suppression mechanism to enhance temporal consistency and suppress background distractors. The modified objective is defined as an energy function:

(1) $$\begin{array}{cc} E(g)=\; & \sum_{c=1}^{C}\|y_{c}-\phi_{c}^{t}★g_{c}{\|}_{2}^{2}+\sum_{c=1}^{C}\|\omega ⊙g_{c}{\|}_{2}^{2}\\ \; & +\lambda \sum_{c=1}^{C}\|R^{t-1}⊙M_{ij}-\left(\psi_{c}^{t}⊙P\right)★g_{c}{\|}_{2}^{2} \end{array}$$

where ★ is the correlation operation, and ⊙ is the element-wise multiplication, and λ denotes the response-interference-suppression penalty factor. To minimize the discrepancy between the predicted response map—obtained by correlating $\phi_{c}^{t}$ (the c-th channel of the target appearance feature extracted from the current frame *t*) with $g_{c}$ (the filter being trained)—and $y_{c}$, a Gaussian-shaped label centered at the target, the objective function performs supervised correlation filter learning. Here, ω represents the penalty regularization weight. In addition, to effectively suppress background distractions, a new part is applied in the objective function. $R^{t-1}$ is the response map from the previous frame, $M_{ij}$ is a binary mask centered at the previous target location (i,j), $\psi_{c}^{t}$ is the background-enhanced feature extracted from the t-th frame, and P is the pixel-wise background weight map in which the value of the background is higher than that of the target.

The solution to (1) is decoupled into *C* independent subproblems by dropping the subscript ${\left(\cdot \right)}_{c}$. Then, in order to facilitate efficient optimization, the objective is reformulated in the frequency domain by adding an auxiliary variable *u* and an equality constraint. The resulting frequency-domain formulation is

(2) $$\begin{array}{cc} \arg\min_{g}\;\; & {∥\hat{y}-{\bar{\hat{\phi }}}^{t}⊙\hat{g}∥}_{2}^{2}+{∥\omega ⊙u∥}_{2}^{2}\\ \; & +\lambda {∥{\hat{R}}_{M}^{t-1}-{\bar{\hat{\psi }}}_{P}^{t}⊙\hat{g}∥}_{2}^{2}\\ s.t.\; & \hat{g}=\sqrt{N}Fu \end{array}$$

where ⌃ denotes the discrete Fourier transform, and ^−^ indicates complex conjugation. The equality constraint $\hat{g}=\sqrt{N}Fu$ ensures that the learned filter in the frequency domain is consistent with its spatial-domain counterpart, and *N* is the size of $\hat{g}$ in a single channel. To solve the constraint problem, the augmented Lagrangian method is adopted, leading to the following objective:

(3) $$\begin{array}{cc} L(\hat{g},\hat{\zeta })=\;\; & {∥\hat{y}-{\bar{\hat{\phi }}}^{t}⊙\hat{g}∥}_{2}^{2}+{∥\omega ⊙u∥}_{2}^{2}\\ \; & +\lambda {∥{\hat{R}}_{M}^{t-1}-{\bar{\hat{\psi }}}_{P}^{t}⊙\hat{g}∥}_{2}^{2}\\ \; & +\mu {∥\hat{g}-\sqrt{N}Fu+\frac{1}{\mu }\hat{\zeta }∥}_{2}^{2} \end{array}$$

where a penalty term and a dual variable λ are incorporated to softly enforce the constraint. $\hat{\zeta }$ acts as a Lagrange multiplier in the frequency domains, and μ is a penalty parameter balancing constraint enforcement and optimization stability. Subsequently, ADMM [15] is used to iteratively solve three subproblems. Firstly, the closed-form solution for solving ${\hat{g}}^{i}$ at iteration i is given below:

(4) $${\hat{g}}^{i}=\frac{{\hat{S}}_{\phi y}+\lambda {\hat{\psi }}_{P}^{t}⊙{\hat{R}}_{M}^{t-1}+\mu \sqrt{N}Fu^{\left(i-1\right)}-{\hat{\zeta }}^{\left(i-1\right)}}{{\hat{S}}_{\phi \phi }+\lambda {\hat{S}}_{\psi \psi }+\mu }$$

where ${\hat{S}}_{\phi y}={\hat{\phi }}^{t}⊙\bar{\hat{y}},\;{\hat{S}}_{\phi \phi }={\hat{\phi }}^{t}⊙{\bar{\hat{\phi }}}^{t},\;{\hat{S}}_{\psi \psi }={\hat{\psi }}_{p}⊙{\bar{\hat{\psi }}}_{p}^{t}$. The term ${\hat{S}}_{\phi y}$ is the cross-correlation between the training feature ${\hat{\phi }}^{t}$ and label $\bar{\hat{y}}$, while ${\hat{S}}_{\phi \phi }$ and ${\hat{S}}_{\psi \psi }$ are the auto-correlations of the target and background-enhanced features, respectively. The penalty term μ ensures convergence and numerical stability. Secondly, the auxiliary variable *u* is updated as follows:

(5) $$u^{\left(i\right)}=\frac{F^{-1}\left(\mu {\hat{g}}^{i}+{\hat{\zeta }}^{\left(i-1\right)}\right)}{\frac{1}{N}\left(\omega ⊙\bar{\omega }\right)+\mu }$$

where $F^{-1}$ represents the inverse discrete Fourier transform. Then, in the i-th iteration, the ${\hat{\zeta }}^{\left(i\right)}$ is updated as follows:

(6) $${\hat{\zeta }}^{\left(i\right)}={\hat{\zeta }}^{\left(i-1\right)}+\mu \left({\hat{g}}^{\left(i\right)}-{\hat{u}}^{\left(i\right)}\right)$$

Here, the penalty parameter $\mu^{i}$ is updated by multiplying the previous value by β and limiting it with a maximum value $\mu_{\max}$, i.e., $\mu^{i}=\min\left\{\mu_{\max},\beta \mu^{i-1}\right\}$ sets the upper bound and β controls the growth rate. Afterward, to account for appearance variation over time, the model template is updated online using an exponential moving average:

(7) $${\hat{\phi }}^{t}=\left(1-\eta \right){\hat{\phi }}^{t-1}+\eta {\hat{\psi }}^{t}$$

Here, η∈[0,1] is the learning rate, determining how much weight is given to new observations versus historical information. This update allows gradual adaptation to new appearance features ${\hat{\psi }}^{t}$ while retaining information from the past template ${\hat{\phi }}^{t-1}$.

Finally, to ensure that only regions near the expected target center receive a high level of attention, a response auxiliary strategy is used to stabilize the response map. The formulation is given as follows:

(8) $$R^{t}=F^{-1}\left(\sum_{c=1}^{C}{\hat{g}}_{c}^{t-1}⊙{\hat{\psi }}_{c}^{t}\right)⊙V$$

Here, $R^{t}$ is the response map modulated by a spatial weighting mask V centered at the previous peak $\left(i_{c},j_{c}\right)$. The weight matrix is defined as $V\left(i,j\right)=\exp\left(\delta \cdot \sqrt{{\left(i-i_{c}\right)}^{2}+{\left(j-j_{c}\right)}^{2}}\right)$, where δ<0 controls the sharpness of decay. The value of the upper bound for the sum sign *C* is the total number of channels of integrated multi-features.

For the more detailed formulation, please refer to [20]. Thanks to its spatial–temporal regularization, adaptive appearance modeling and spatial response smoothing, the learned filter can become more robust and generalized. Meanwhile, many effective acceleration techniques are adapted to reduce the computational cost of real-time tracking when solving the response-interference-suppression correlation filter. However, since multiple features and a spatial–temporal scale space are adapted to cope with target-state estimation, straightforward operation with fixed feature dimensions and dense scale intervals may be computationally expensive and suboptimal for handling large-scale changes in the target in the RISTrack framework. Contrary to the RISTrack method, PSST [27] employs a robust state prediction scheme using a particle filter framework with inherent state estimation and performs favorably compared to state-of-the-art methods. However, this scheme is not suitable for fast state estimation, since a large number of samples are used to ensure tracking accuracy in spatial–temporal domains. To improve upon the above state estimation methods, we propose an adaptive feature response and scale-interval method with a histogram score map to reduce the feature dimensions and the frequency of scale estimation when updating the target location and scale.

### 3.2. Adaptive Mapped-Feature Response

Multiple-feature integration is often employed to boost tracking performance [20]. However, this straightforward operation, which involves simply concatenating features, is not universally applicable for enhancing tracking precision, and may result in tracking performance degradation [15]. To avoid degradation during multiple-feature integration, an adaptive mapped-feature response based on dimensionality reduction and histogram-based score mapping is proposed to improve tracking ability. Specifically, we first adaptively select the optimal dimensions of the integrated features to maintaining the real-time characteristics of RISTrack. Then, the features of the selected dimensions, visualized with an adaptive color map, which is based on the histogram-based per-pixel score, are used to generate the final responses and enhance the robustness of the state estimation. We apply three commonly used features, including the histogram of oriented gradient (HOG), color names (CNs) [23] and a color map (CM) [28], in the adaptive mapped-feature response, where a CM is applied to HOG+CN features. Therefore, a translation filter and a histogram model from [28] are trained for the response maps of different features, where ${\hat{g}}_{c}$ denotes the HOG+CN-based filter, and *h* represents the histogram model for CM. For more detailed histogram model formulas, please refer to [28].

The dimensionality reduction technique is based on the standard principal component analysis applied in the adaptive mapped-feature response approach. The updated model template on the HOG+CN feature is defined as $u^{\left(t\right)}=\left(1-\eta \right)u^{\left(t-1\right)}+\eta \psi^{\left(t\right)}$, which is employed to adaptively reduce the feature dimensions. Here, $u^{\left(0\right)}$ is zero, and $u^{\left(1\right)}$ represents the extracted HOG+CN features in the t=1 frame. Then, Equation (7) can be reformulated as $F\left\{u^{\left(t\right)}\right\}=\left(1-\eta \right)F\left\{u^{\left(t-1\right)}\right\}+\eta {\hat{\psi }}^{\left(t\right)}$ by the linear Fourier transform ${\hat{\phi }}^{\left(t\right)}=F\left\{u^{\left(t\right)}\right\}$. Finally, the updated model template $u^{\left(t\right)}$ can be adopted to construct a projection matrix $P^{\left(t\right)}$, which is the low-dimensional subspace. The features can be projected onto this subspace by the projection matrix $P^{\left(t\right)}$, which has a size of $\tilde{c}\times C$, where $\tilde{c}$ is the dimension of the compressed feature. The projection matrix $P^{\left(t\right)}$ is obtained by minimizing the objective function for reconstructing the updated model template $u^{\left(t\right)}$:

(9) $$\begin{array}{cc} \varepsilon =\sum_{m}\|u^{\left(t\right)} & \left(m\right)-P^{(t)T}P^{\left(t\right)}u^{\left(t\right)}{\left(m\right)\|}^{2}\\ \; & s.t.\;P^{\left(t\right)}P^{(t)T}=I \end{array}$$

where m denotes the index tuple ranging over all elements in the model template $u^{\left(t\right)}$. Then, we obtain a solution through eigenvalue decomposition of the auto-correlation matrix for $u^{\left(t\right)}$:

(10) $$C^{\left(t\right)}=\sum_{m}u^{\left(t\right)}\left(m\right)u^{\left(t\right)}{\left(m\right)}^{T}$$

We set the rows of $P^{\left(t\right)}$ to the $\tilde{k}$ eigenvectors of $C^{\left(t\right)}$ with the largest eigenvalues. The target patch is updated using the compressed model template ${\hat{U}}^{\left(t\right)}=F\left\{P^{\left(t\right)}u^{\left(t\right)}\right\}$ as follows:

(11) $${\hat{\phi }}^{\left(t\right)}={\hat{U}}_{l}^{\left(t\right)},\;\;l=1,....,\tilde{c}$$

We employ the linear operation of $P^{\left(t\right)}$ as an element-wise matrix multiplication $\left(P^{\left(t\right)}u^{\left(t\right)}\right)\left(m\right)=P^{\left(t\right)}u^{\left(t\right)}\left(m\right)$, which projects the feature vector $u^{\left(t\right)}\left(m\right)\in R^{C}$ onto the rows of $P^{\left(t\right)}$. During the detection stage, the response maps in the model template ${\hat{\psi }}^{\left(t\right)}$ are computed by applying the HOG+CN feature-based filter to the compressed template ${\hat{\psi }}^{\left(t\right)}=F\left\{P^{\left(t-1\right)}{\hat{\psi }}^{\left(t\right)}\right\}$, similarly to Equation (8):

(12) $$R^{t}=F^{-1}\left(\sum_{c=1}^{\tilde{c}}{\hat{g}}_{c}^{t-1}⊙{\hat{\psi }}_{c}^{t}\right)⊙V$$

Similarly to [20], we use the Hann window *W* to avoid boundary effects. Furthermore, inspired by [28], the adaptive map with color information is applied to the adaptive-dimension features in a simple way to generate the final accurate responses: $\psi^{\prime }=\psi ⊙\left\{\chi \left(H⊙W\right)+\left(1-\chi \right)W\right\}$; here, χ is the adaptive map value, $\psi^{\prime }$ represents the adaptive mapped features with dimensionality reduction, and *H* is the adaptive color map obtained by computing the per-pixel score of the target template with the histogram model *h* [28].

### 3.3. Adaptive Temporal Scale-Interval Estimation

Most DCF trackers are primarily used to estimate the scales of searching areas at multiple resolutions, as is the case for STRCF [24]. They are also used to predict size by employing dedicated scale-space filters such as RISTrack [20]. However, these approaches are limited to dense spatial–temporal scale-space sampling to handle the large-scale variations instead of adaptive temporal-scale intervals, and may therefore be suboptimal. Most of the remaining state-of-the-art methods employ a particle filter framework to implement robust scale estimation [27]; however, they incur increased computational costs and are not suitable for efficient scale estimation, since a large number of samples are used in spatial–temporal domains to ensure tracking accuracy. This makes them infeasible for real-time IoT applications. Consequently, scale estimation should not only be robust to changes in the target scale, but it should also take the computational efficiency into account.

In light of this, it would be helpful to explore adaptive temporal scale estimation to reduce the number of video frames with scale estimation to achieve a trade-off between effectiveness and efficiency. We assume T to represent the scale intervals in the temporal domains, and it is set to a value of one in most trackers to apply a scale filter to each video frame [11,16,20]. Unlike these trackers, for the proposed tracker, the video frames where the target sizes should be estimated are selected based on the adaptive temporal scale intervals $C=\bar{s}T$ (let $\bar{s}$ denote the frequency of switching the scale filter).It is worth noting that in addition to the above adaptive scale intervals in the temporal domain, we always use original fixed scale strides in the spatial domain to ensure tracking stability [11,16,20]. The scale filter is then applied to the selected video frames with adaptive temporal scale intervals to cope with the target sizes. Even though the appropriate scale may not be included in the selected video frames with adaptive scale intervals in the temporal domain, it is likely that the appropriate scale can be determined using numerous adaptive scale intervals of the following selected video frames according to the common smallness and smoothness of target scale variations across all video frames. Our experiments in Section 4.2.4 also demonstrate that our scale estimation with adaptive intervals in the temporal domain significantly improve tracking efficiency while maintaining almost identical accuracy with sparse scale intervals.

### 3.4. Tracking

Combining the response-interference-suppression correlation filter [20], adaptive mapped-feature response based on dimensionality reduction and histogram score maps, and adaptive temporal scale-interval estimation, we propose ARIST, a flowchart for which is shown in Figure 2. The whole tracking process is divided into two parts: filter training and target prediction. During the training and updating stage, a translation filter based on adaptive dimension features is trained by suppressing response interference, similarly to RISTrack [20]. In addition, the scale filter based on the adaptive temporal scale interval and the color map-based histogram model are also prepared in parallel [20,28]. During the target prediction stage, the translation filter with the adaptive dimension features and the histogram model with the adaptive maps are first applied to predict the target location using Equation (12). The scale filter with the adaptive temporal scale intervals is then adopted to compute the final target sizes.

## 4. Experiments

To fully assess the effectiveness of the proposed methods, we conduct experiments on four standard benchmark datasets: UAV123@10fps [4], DTB70 [21], UAV112 [3] and UAVDT [22]. These benchmarks involve video sequences with various challenges, including fast motion (FM), motion blur (MB), in-plane rotation (IPR) and illumination variation (IV). Specifically, UAV123@10fps is built by downsampling 123 videos of UAV123 [4] at 10 FPS, thus leading to more challenging scenarios, like fast motion and motion blur. DTB70 consists of 70 videos collected by a UAV and has a total of 15,777 frames. UAV112 includes 112 low-altitude aerial videos with fast motion, long-term tracking and low resolution. Similarly to DTB70, UAVDT includes 100 videos captured by a UAV in complex environments. We follow the protocols and standard evaluation metrics in these datasets, and the precision and success scores are adapted in our evaluation. Precision scores measure the number of frames whose center is within 20 pixels of ground-truth positions. Success scores measure the percentage of overlap between the tracking results and ground truth boxes. Further, we employ the one-pass evaluation (OPE) in all of our evaluations.

### 4.1. Implementation Details

The proposed trackers were executed in MATLAB 2017a without any optimization. All of the experiments were implemented on a PC with an Intel Core i7-1165G7 CPU (2.80 GHz) and 16 GB of RAM. On DTB70, ARIST runs at about 41.3 FPS. The cell size used in HOG is 4 × 4, and the orientation bin number is 9. The adaptive-dimension feature coefficient $\tilde{c}$ is 23; the adaptive mapped fusion parameter χ is 0.89; and the frequency $\bar{s}$ of switching the scale filter in the adaptive temporal scale intervals is set to 2. For more detailed information on the parameter settings, please refer to [20,28].

### 4.2. Ablation Study of ARIST

In this section, we present the ablation study and the precision and success scores on DTB70.

#### 4.2.1. Component Analysis of ARIST

Theoretically, ARIST’s tracking performance is primarily related to the adaptive mapped-feature response and adaptive temporal scale-interval estimation; thus, we implement four more algorithms to demonstrate its effectiveness. First, we implement RISTrack [20], with the fixed features and scale as the baseline; second, ARIST-AF is implemented, with only an adaptive mapped-feature response; third, ARIST-AS is employed using only adaptive temporal scale-interval estimation with color maps; and fourth, we construct RISTrack-PSST by employing a particle scale space [27]. According to Table 1, ARIST achieves the best results among these trackers. Specifically, ARIST-AF achieves precision and success scores about 1.0% and 0.3% higher than those of RISTrack, which verifies the effectiveness of the adaptive mapped-feature response based on dimensionality reduction and histogram score maps. Compared to RISTrack, ARIST-AS and ARIST achieve better performance by considering scale estimation in different ways. Among these scale trackers, ARIST has precision and success scores 0.3% and 0.5% higher than those of ARIST-AF. This illustrates that the proposed adaptive temporal scale-interval estimation is efficient. In addition, ARIST has precision and success scores 1.1% and 1.1% higher than those of RISTrack-PSST, which also verifies the superiority of our temporal sparse scale intervals. Most notably, compared with RISTrack, ARIST achieves 1.3% and 0.8% higher precision and success scores, respectively, with a modest impact on efficiency.

#### 4.2.2. Adaptive Map Value Analysis

**Figure 3:**
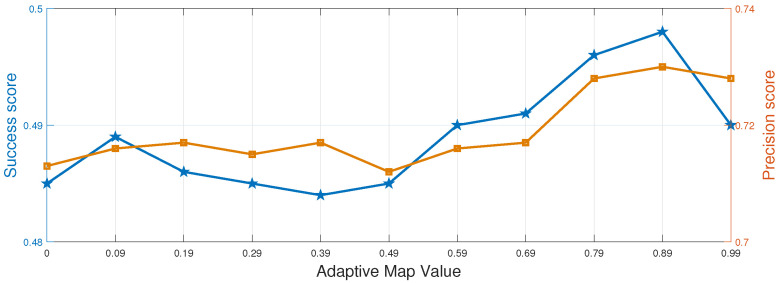
Adaptive map values χ of ARIST, showing the success (blue line) and precision (orange line) scores in the OPE for the DTB70 dataset.

As described in Section 3.2, we employ an adaptive mapped-feature response based on dimensionality reduction and histogram scores in ARIST. We analyze the adaptive map values χ in multiple features on DTB70. Figure 3 shows the tracking performance, in terms of precision and success scores, throughout the OPE for different adaptive map values. We observe that the performance of ARIST is inferior to that of the state-of-the-art trackers when using only the fixed Hann window.However, the precision and success scores of ARIST continue to improve with increasing adaptive map values of the histogram scores, until a histogram score-based adaptive map value χ of 0.89 is reached. We set the adaptive map value to 0.89 between the Hann window and the adaptive color map for all of our experiments.

#### 4.2.3. Feature Dimension Analysis of ARIST

**Figure 4:**
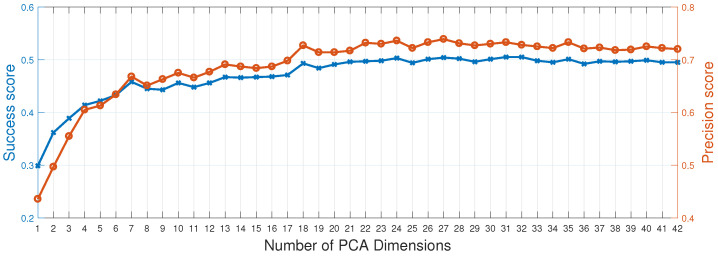
Impact of the number of feature dimensions in ARIST. The tracking performance on DTB70, in terms of precision (orange line) and success (blue line) scores, is reported for different numbers of dimensions $\tilde{c}$.

As described in Section 3.2, we also employ a dimensionality reduction scheme for the adaptive mapped-feature response. Therefore, we analyze the impact of varying the number of HOG+CN feature dimensions in ARIST. Figure 4 shows the tracking performance, in terms of precision and success scores, obtained during the OPE on DTB70 for different numbers of feature dimensions. The performance of ARIST remains largely consistent when the number of dimensions is reduced from 42, and then degrades rapidly at about 18. Our results suggest that the feature dimensions can be significantly reduced with our framework while maintaining comparable tracking performance to state-of-the-art trackers. To achieve a trade-off between efficiency and effectiveness, we set the number of HOG+CN feature dimensions to 23 in ARIST. Overall, compared to the original feature extraction method, with 42 dimensions running at about 33.5 FPS, our feature dimension reduction method, with 23 dimensions running at about 41.3 FPS, achieves a 23% improvement in tracking efficiency.

#### 4.2.4. Adaptive Temporal Scale-Interval Analysis of ARIST

As shown in Section 3.3, efficient scale estimation is achieved by increasing the number of temporal scale intervals C. Thanks to its temporal adaptive scale intervals, ARIST can adopt sparse scale intervals to handle the target scale changes, unlike RISTrack. As shown in Table 2, using only two scale intervals can enhance tracking performance by handling scale changes, which is not the case when one scale interval is used. It is also observed that increasing the number of temporal scale intervals can improve tracking efficiency, but tracking performance will be reduced. Here, setting the temporal scale interval to two can effectively achieve a trade-off between accuracy and efficiency. In addition, since tracking performance is sensitive to the frequency $\bar{s}$ of switching the scale filter, it is unstable after reaching its optimal value.

### 4.3. Comparison with State-of-the-Art Trackers

In this section, we evaluate ARIST in comparison to the most recent state-of-the-art trackers, including handcrafted feature trackers (SRECF [19], AutoTrack [25], ARCFH [5], STRCF [24], L1CFT [11], BACF [15], ECO-HC [26], MRCF [14], RISTrack [20] and EMCF [17]) and deep learning trackers (LUDT [29], MCCT [30], ASRCF [18], STARK-st101 [8], HiFT [7], TCTrack [6], SGDViT [10] and AVTrack [9]).

#### 4.3.1. Comparison with Handcrafted Feature Trackers

**Figure 5:**
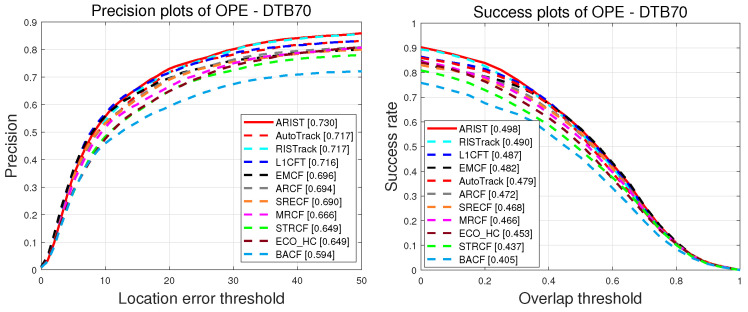
Plots of precision and success of handcrafted feature trackers in the OPE on DTB70.

**DTB70.** Figure 5 shows the precision and success scores throughout the OPE on DTB70 [21]. Regarding the success scores, the proposed tracker outperforms the other state-of-the-art trackers, achieving success and precision scores 1.1% and 1.3%, respectively, higher than those of L1CFT, which was recently proposed in combination with a sparse regularization-enhanced correlation filter for UAV tracking. Furthermore, ARIST is 1.3% and 1.3% better than the recent AutoTrack and RISTrack, respectively, in terms of precision scores. Regarding precision and success scores obtained during the OPE on DTB70, the proposed tracker ranks at the top, achieving superior performance compared with the state-of-the-art trackers.

**Figure 6:**
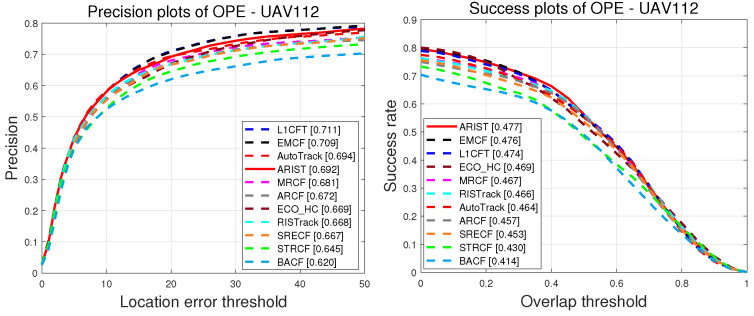
Plots of precision and success of handcrafted feature trackers in OPE on UAV112.

**UAV112.** The precision and success scores obtained during the OPE on UAV112 [3] are shown in Figure 6. It is evident that although the precision scores of ARIST are lower than those of AutoTrack, EMCF and L1CFT, the reported running times and success scores of the latter suffer as these trackers use more dimensional features and temporal scale intervals.Furthermore, ARIST, with precision and success scores of 69.2% and 47.7%, outperforms ECO_HC, ARCF, RISTrack, BACF, STRCF, SRECF and MRCF. Overall, the proposed tracker achieves comparable tracking performance to the state-of-the-art trackers.

**Figure 7:**
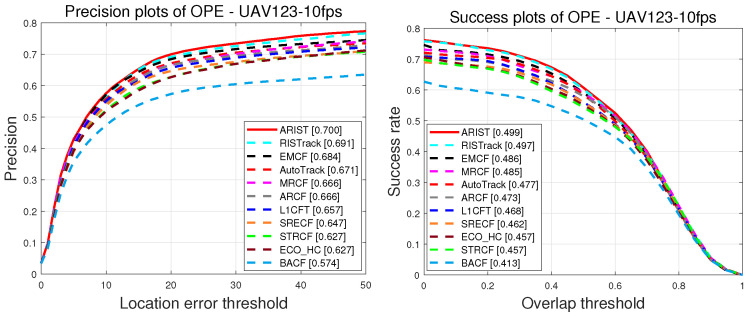
Plots of precision and success of handcrafted feature trackers in OPE on UAV123@10fps.

**UAV123@10fps.** Figure 7 shows the precision and success scores obtained during the OPE on UAV123@10fps [4]. We can see that ARIST performs favorably compared to the state-of-the-art trackers, with precision and success scores of 70.0% and 49.9%, which is basically consistent with the evaluation results for the above datasets. Specifically, ARIST obtains success scores 2.2%, 1.3% and 3.1% higher than those of AutoTrack, EMCF and L1CFT, which all achieve superior performance in UAV112. More specifically, ARIST runs at a real-time speed of 41.3 FPS on a CPU.

**Figure 8:**
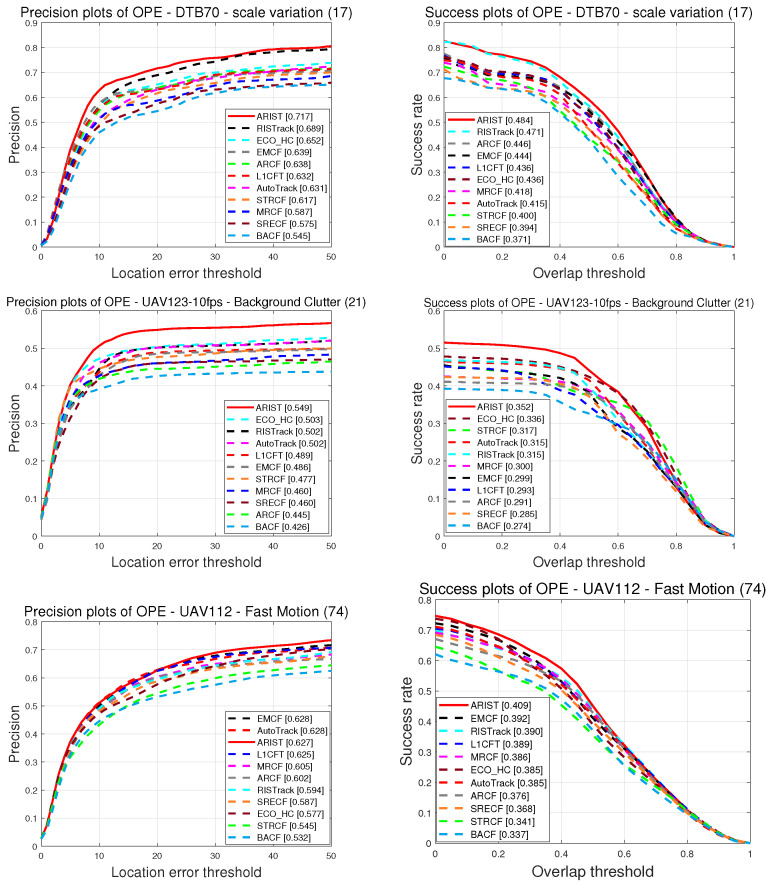

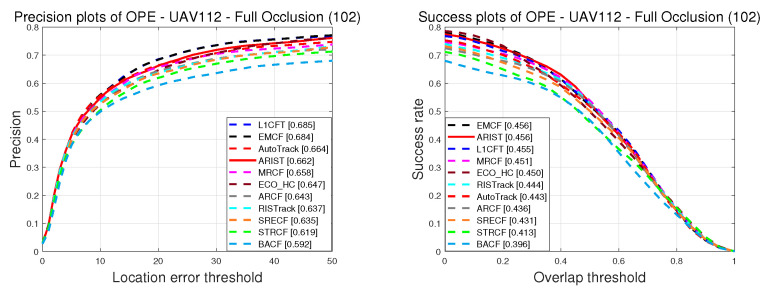
Plots of precision and success of handcrafted feature trackers for four attributes, including scale variation in DTB70, background clutter in UAV123@10fps, and fast motion and full occlusion in UAV112.

**Attributeanalysis.** For a more comprehensive evaluation of ARIST, Figure 8 shows the tracking results for the four most challenging attributes according to the OPE on DTB70, UAV112 and UAV123@10fps, with ARIST ranking among the top four. Among the existing methods, the RISTrack method performs well in terms of scale variation with regard to the precision (68.9%) and success (47.1%) scores, while the proposed ARIST algorithm achieves scores of 71.7% and 48.4%, respectively. The ECO_HC method performs well in terms of background clutter with regard to the precision (50.3%) and success (33.6%) scores, while the ARIST algorithm achieves scores of 54.9% and 35.2%. In terms of fast motion and full occlusion, ARIST achieves comparable results to EMCF and AutoTrack, which use more dimensional features and temporal scale intervals.

#### 4.3.2. Comparison with Deep Learning Trackers

To further validate the effectiveness of the proposed ARIST algorithm, it is evaluated against the UAVDT benchmark [22] with recent state-of-the-art trackers based on deep learning. As shown in Table 3, compared to the state-of-the-art deep learning-based trackers (SGDViT, TCTrack, HiFT, STARK-st101, LUDT, ASRCF and MCCT), which rely on high-end GPUs, ARIST outperforms in terms of precision scores. However, the proposed tracker achieves lower success scores than TCTrack and AVTrack because of the limited capacity of handcrafted features.

## 5. Conclusions

In this paper, we propose an adaptive mapped-feature and scale-interval method based on a response-interference-suppression correlation filter to overcome the problem of fixed feature dimensions and dense spatial–temporal scale-space sampling. Adaptive temporal scale estimation and an adaptive mapped-feature response based on dimensionality reduction with histogram score maps are proposed to reduce the number of dimensional features and temporal scale intervals and enhance target-state estimation in terms of both effectiveness and efficiency. Extensive evaluations on the UAV123@10fps, DTB70, UAV112 and UAVDT datasets clearly demonstrate that the proposed tracker exhibits improved performance while being computationally efficient compared to the baseline tracker with fixed features and dense scale intervals. The tracker also performs favorably compared to state-of-the-art trackers while operating in real time, which makes it suitable for resource-constrained end devices in various IoT applications. Future studies will include optimizing the tracker for speed and edge deployment (e.g., NanoTrack and DiMP-light) and strengthening its suitability for actual IoT hardware platforms (e.g., Raspberry Pi, NVIDIA Jetson and Embedded UAV Systems).

## Figures and Tables

**Table 1:** Comparisons of component effectiveness on DTB70 in OPE. The best results are displayed in red.

Tracker	RISTrack	RISTrack-PSST	ARIST-AF	ARIST-AS	ARIST
Precision	0.717	0.719	0.727	0.720	0.730
Success	0.490	0.487	0.493	0.495	0.498
FPS	42.1	37.6	36.5	33.5	41.3

**Table 2:** Results of the evaluation of ARIST with respect to temporal scale intervals on DTB70, reported as precision scores, success scores and mean FPS. The best results are bolded.

Temporal Scale Interval	1	2	3	4	5
Precision/Success	0.727/0.493	**0.730/0.498**	0.730/0.490	0.717/0.481	0.732/0.488
Mean FPS	36.5	**41.3**	42.3	43.1	44.3

**Table 3:** Precision and success scores of deep learning trackers in OPE on UAVDT. The best and second-best scores are presented in red and green, respectively. The * means GPU speed.

Tracker	MCCT [30]	ASRCF [18]	LUDT [29]	STARK-st101 [8]	HiFT [7]	TCTrack [6]	SGDViT [10]	AVTrack [9]	ARIST
Venue	CVPR’18	CVPR’19	IJCV’21	ICCV’21	ICCV’21	CVPR’22	ICRA’23	ICML’24	Ours
Precision	0.671	0.700	0.701	0.704	0.652	0.725	0.657	0.788	0.745
Success	0.437	0.469	0.406	0.469	0.475	0.530	0.480	0.572	0.470
FPS	8.8 *	22.3 *	59.3 *	37.9 *	-	-	-	-	41.3

## Data Availability

No new datasets were created in this study.

## References

[1] Cao W., Zhang J., Cai C., Chen Q., Zhao Y., Lou Y., Jiang W., Gui G. (2021). CNN-based intelligent safety surveillance in green IoT applications. China Commun..

[2] Smeulders A., Chu D., Cucchiara R., Calderara S., Deghan A., Shah M. (2014). Visual tracking: An experimental survey. IEEE Trans. Pattern Anal. Mach. Intell..

[3] Fu C., Cao Z., Li Y., Ye J., Feng C. (2021). Onboard real-time aerial tracking with efficient Siamese anchor proposal network. IEEE Trans. Geosci. Remote Sens..

[4] Mueller M., Smith N., Ghanem B. A Benchmark and Simulator for UAV Tracking. Proceedings of the European Conference on Computer Vision.

[5] Huang Z., Fu C., Li Y., Lin F., Lu P. Learning Aberrance Repressed Correlation Filters for Real-Time UAV Tracking. Proceedings of the IEEE International Conference on Computer Vision.

[6] Cao Z., Huang Z., Pan L., Zhang S., Liu Z., Fu C. TCTrack: Temporal contexts for aerial tracking. Proceedings of the IEEE/CVF Conference on Computer Vision and Pattern Recognition.

[7] Cao Z., Fu C., Ye J., Li B., Li Y. HiFT: Hierarchical feature transformer for aerial tracking. Proceedings of the IEEE/CVF International Conference on Computer Vision.

[8] Yan B., Peng H., Fu J., Wang D., Lu H. Learning spatio-temporal transformer for visual tracking. Proceedings of the IEEE/CVF InternationalConference on Computer Vision.

[9] Li Y., Liu M., Wu Y., Wang X., Yang X., Li S. Learning adaptive and view-invariant vision transformer for real-time UAV tracking. Proceedings of the Forty-First International Conference on Machine Learning.

[10] Yao L., Fu C., Li S., Zheng G., Ye J. SGDViT: Saliency-guided dynamic vision transformer for UAV tracking. Proceedings of the 2023 IEEE International Conference on Robotics and Automation.

[11] Ji Z., Feng K., Qian Y., Liang J. (2024). Sparse regularized correlation filter for UAV object tracking with adaptive contextual learning and keyfilter selection. Inf. Sci..

[12] Bolme D.S., Beveridge J.R., Draper B.A., Lui Y.M. Visual object tracking using adaptive correlation filters. Proceedings of the IEEE Conference on Computer Vision and Pattern Recognition.

[13] Henriques J.F., Caseiro R., Martins P., Batista J. (2015). Highspeed tracking with kernelized correlation filters. IEEE Trans. Pattern Anal. Mach. Intell..

[14] Ye J., Fu C., Lin F., Ding F., An S., Lu G. (2021). Multi-regularized correlation filter for UAV tracking and self-localization. IEEE Trans. Ind. Electron..

[15] Galoogahi H.K., Fagg A., Lucey S. Learning Background-Aware Correlation Filters for Visual Tracking. Proceedings of the IEEE International Conference on Computer Vision.

[16] Chen Z., Liu L., Yu Z. (2025). Learning Dynamic Distractor-Repressed Correlation Filter for Real-Time UAV Tracking. IEEE Signal Process. Lett..

[17] Zhang F., Ma S., Zhang Y., Qiu Z. (2021). Perceiving temporal environment for correlation filters in real-time UAV tracking. IEEE Signal Process. Lett..

[18] Dai K., Wang D., Lu H., Sun C., Li J. Visual Tracking via Adaptive Spatially-Regularized Correlation Filters. Proceedings of the IEEE Conference on Computer Vision and Pattern Recognition.

[19] Fu C., Jin J., Ding F., Li Y., Lu G. (2021). Spatial Reliability Enhanced Correlation Filter: An Efficient Approach for Real-Time UAV Tracking. IEEE Trans. Multimedia.

[20] Li Y., Zhang H., Yang Y., Liu H., Yuan D. (2023). RISTrack: Learning response interference suppression correlation filters for UAV tracking. IEEE Geosci. Remote Sens. Lett..

[21] Li S., Yeung D. Visual object tracking for unmanned aerial vehicles: A benchmark and new motion models. Proceedings of the Thirty-first AAAI Conference on Artificial Intelligence.

[22] Yu H., Li G., Zhang W., Huang Q., Du D., Tian Q., Sebe N. (2020). The unmanned aerial vehicle benchmark: Object detection, tracking and baseline. Int. J. Comput. Vis..

[23] Danelljan M., Khan F.S., Felsberg M., van de Weijer J. Adaptive color attributes for real-time visual tracking. Proceedings of the IEEE Conference on Computer Vision and Pattern Recognition.

[24] Li F., Tian C., Zuo W., Zhang L., Yang M.H. Learning Spatial-Temporal Regularized Correlation Filters for Visual Tracking. Proceedings of the IEEE Conference on Computer Vision and Pattern Recognition.

[25] Li Y., Fu C., Ding F., Huang Z., Lu G. AutoTrack: Towards High-Performance Visual Tracking for UAV with Automatic Spatio-Temporal Regularization. Proceedings of the IEEE Conference on Computer Vision and Pattern Recognition.

[26] Danelljan M., Bhat G., Khan F.S., Felsberg M. ECO: Efficient Convolution Operators for Tracking. Proceedings of the IEEE Conference on Computer Vision and Pattern Recognition.

[27] Li S., Zhao S., Cheng B., Chen J. (2020). Efficient Particle Scale Space for Robust Tracking. IEEE Signal Process. Lett..

[28] Bertinetto L., Valmadre J., Golodetz S., Miksik O., Torr P.H.S. Staple: Complementary learners for real-time tracking. Proceedings of the IEEE Conference on Computer Vision and Pattern Recognition.

[29] Wang N., Zhou W., Song Y., Ma C., Liu W., Li H. (2021). Unsupervised deep representation learning for real-time tracking. Int. J. Comput. Vis..

[30] Wang N., Zhou W., Tian Q., Hong R., Wang M., Li H. Multi-Cue Correlation Filters for Robust Visual Tracking. Proceedings of the IEEE Conference on Computer Vision and Pattern Recognition.

